# (Case Of) Hypospadias—Operation—Remarkable Result

**Published:** 1874-01

**Authors:** 


*RICHMOND AND LOUISVILLE MED. JOURNAL--November*, 1873:

A Case of Hypospadias—Operation—Recovery.—*By Drs. Ishmael and Browne.*—Mrs. H., aged twenty-eight years, married five years; childless; applied to us for medical treatment on the 3d of November, 1872, complaining in such a manner and of such ailments, that we diagnosed the case to be one of chronic cervical endometritis—the first symptoms of which were noticed a few months after marriage, increasing in gravity up to the date of this consultation, notwithstanding she had been under the care of reputable physicians all the time.

On inquiry we learned that her husband, the subject of this article, aged forty-four years, and a man of the most perfect apparent health, had been married prior to the union with his present wife. His first wife lived seven years after marriage, and died childless. The disease of which she died ran a similar course to that of his present wife, which we, from the subseqnent history of the case, suppose to have been chronic cervical endometritis, passing in time into corporeal endometritis, and finally destroying the patient by supervention of acute ovaritis, going on to suppuration accompanied by pelvic cellulitis.

We thought this something more than a mere coincidence, and in search for the cause, found the husband, T. H., laboring under a malformation of the penis—hypospadias.

The urethral opening was discovered about the anterior part of the membranous portion of the urethra; the scrotum cleft in the median line. The glans penis, corona glandis, prepuce, and frænum perputii were naturally formed, with the usual vertical fissure at summit of the glans, but no trace of meatus urinarius.

Believing that we had discovered the cause producing the disorders of which Mrs. H. was a subject in the husband's infirmity, we suggested an operation for the relief of this malformation, as the only rational treatment we could apply for the relief of the uterine disorder—the cause being removed, the effect would cease.

Having decided upon operating—everything being in readiness; anæsthesia produced by chloroform—we proceeded to the performance of the operation by plunging, not without difficulty, a large size trocar, entering at the usual site of the meatus urinarius through the corpus spongiosum, following as nearly as possible the natural course of the urethra, until the trocar made its appearance in the membranous portion of the urethra, posterior to the fissured outlet.

Removing the trocar, we introduced a large size catheter into the bladder, and united the fissure by means of interrupted sutures, the edges having been pared. The canal was kept pervious by the almost constant wearing of a catheter, until a mucous lining was formed; since which time we are having the catheter worn for a few hours daily, to prevent a contraction of the artificial canal.

The old fissure closed kindly, and the operation speedily terminated in success.

Since operating, we have had the satisfaction of seeing our original patient, Mrs. H., steadily improve, until she now feels entirely well, and is, at this time, in the fourth month of pregnancy. The result demonstrates the correctness of our first diagnosis, prognosis, etiology, etc.

How great is the caution necessary on the part of the physician in searching out the remote causes of disease!

